# How Knowledge Becomes Credible Under Epistemic Uncertainty: A Grounded Theory of Telepathic Animal Communication Websites

**DOI:** 10.1111/cars.70055

**Published:** 2026-09-23

**Authors:** Annika Barzen, Dylan Göpfert

**Affiliations:** ^1^ Chemnitz University of Technology Chemnitz Germany

**Keywords:** document analysis, grounded theory, knowledge construction, sociology of knowledge, telepathic animal communication, websites

## Abstract

How credibility is constructed under conditions of epistemic uncertainty is a central question in the sociology of knowledge. Telepathic animal communication (TAC) represents a field in which knowledge claims are inherently non‐verifiable, yet services are actively marketed and consumed. This study examines how TAC practitioners construct credibility on their websites. Drawing on Grounded Theory, websites offering TAC services in Germany were qualitatively analyzed. Findings identify three central mechanisms of credibility construction. First, practitioners employ professionalized presentations, including structured services, therapeutic terminology, and biographical self‐legitimation. Second, credibility is reinforced through emotional and relational appeal, including personalized communication, expressions of care, and perceived closeness between practitioner and client. Third, strategic non‐verifiability emerges as a distinctive mechanism: the inherent unverifiability of TAC is actively mobilized to protect claims from falsification through interpretative flexibility. The findings show how credibility can be stabilized through professional, relational, and epistemic strategies despite the absence of empirical validation.

## RÉSUMÉ

La manière dont la crédibilité se construit dans un contexte d'incertitude épistémique constitue une question centrale en sociologie de la connaissance. La communication animale par télépathie (CAT) représente un domaine dans lequel les affirmations relatives aux connaissances sont intrinsèquement invérifiables, alors que les services sont activement commercialisés et consommés. Cette étude examine comment les praticiens de la CAT construisent leur crédibilité sur leurs sites web. En s'appuyant sur la théorie ancrée, les sites web proposant des services de CAT en Allemagne ont fait l'objet d'une analyse qualitative. Les résultats mettent en évidence trois mécanismes centraux de construction de la crédibilité. Premièrement, les praticiens ont recours à des présentations professionnelles, comprenant des services structurés, une terminologie thérapeutique et une auto‐légitimation biographique. Deuxièmement, la crédibilité est renforcée par un appel émotionnel et relationnel, notamment une communication personnalisée, des manifestations d'attention et une proximité perçue entre le praticien et le client. Troisièmement, la non‐vérifiabilité stratégique apparaît comme un mécanisme distinctif: la non‐vérifiabilité inhérente à la TAC est activement mobilisée pour protéger les affirmations contre toute falsification grâce à une flexibilité interprétative. Les résultats montrent comment la crédibilité peut être stabilisée par le biais de stratégies professionnelles, relationnelles et épistémiques malgré l'absence de validation empirique.

## 1 Introduction

This study addresses a central question in the sociology of knowledge: how credibility is socially constructed and stabilized in contexts where knowledge claims cannot be empirically verified (Berger and Luckmann 1966). Telepathic animal communication (TAC) offers an empirical case with potential for a theoretical contribution to this field, as its claims are inherently non‐verifiable. Individuals who engage with such services cannot rely on independent verification, making TAC a particularly revealing case for examining how credibility is constructed under conditions of epistemic uncertainty.

Recent scholarship on non‐verifiable knowledge has increasingly focused on processes of knowledge production and credibility formation in online environments (Neuberger et al. 2023). Particular attention has been paid to the spread and acceptance of misinformation (Pennycook and Rand 2019) as well as implausible conspiracy narratives (Harambam & Aupers 2015). Across these contexts, credibility is often established in the absence of direct verification through mechanisms such as relational trust (Dahlgren 2018), ideological alignment (Billard and Moran 2023), or appeals to epistemic authority (Parada‐Ulloa et al. 2026). Previous research suggests that situations of uncertainty may increase openness to alternative forms of knowledge (Kelly 2004; Zimmermann et al. 2024).

Recent research has shown that credibility in online contexts is not primarily grounded in empirical validation, but is often produced through relational, affective, and discursive strategies that establish epistemic authority in the absence of verifiable evidence. Building on this perspective, Barzen (2025) demonstrated in the context of YouTube conspiracy discourse how collective epistemic authority can emerge through emotionally engaged communities, where trust and legitimacy are co‐produced between content creators and audiences.

However, less is known about how such processes unfold in settings without interactive audience structures. It also remains unclear how the credibility of unverifiable knowledge is constructed when epistemic claims are embedded in market‐based service relations. Addressing this gap, the present study investigates TAC as an empirical case of non‐falsifiable knowledge claims in a service context. The subject of investigation is addressed with the following research question:

*How do Practitioners of Telepathic Animal Communication construct Credibility regarding their Knowledge and Abilities on their websites?*

To understand how these forms of non‐falsifiable knowledge are credibly communicated, we conducted a qualitative analysis of selected websites of animal communicators. Websites are particularly suitable for this purpose, as they function as primary sites where practitioners describe and actively market their services.

TAC is attracting an increasingly large interest in research focusing on this phenomenon (Sebeok 2011). One such project called “Animal Communicators: Intuitive communication as a key to dialogic multispecies methods” (European Research Council 2024) is currently still in progress. “The first experimental studies on telepathic‐type links between humans and other animals date back to the 1880s, during the rise of meta‐ and para‐psychology” (Monvoisin et al. 2024, 2). For this paper, we understand TAC (also: Intuitive Animal Communication) “as the process of transmitting and receiving information between humans and other animals while transcending conventional communication channels such as explicit verbal, sound, or body language, and involving “intuitive”, “extrasensory”, or even “telepathic” transmission” (ibid.).

TAC is practiced by so‐called “animal communicators” (Barrett et al. 2021, 50), who display certain overarching principles and workflows. Basically, animal communicators “engage in translation efforts between human and non‐human animals” (Wijngaarden 2024, 362). Their work aims “to assist in understanding animals’ perspectives on behavioral, health and other concerns, and to assist in resolving conflicts between animals and people” (Barrett et al. 2021, 154).

A fundamental principle of TAC is the idea of an “Edenic fall” (Trachsel 2022, 92), which frames interspecies communication as a capacity that humans have lost and can relearn. Practitioners often position humans as having become disconnected from interspecies communication, while alternative epistemologies, sometimes drawing on Indigenous knowledge, are framed as pathways to re‐establishing this capacity (Barrett et al. 2021; Wijngaarden 2024).

In preparation for a TAC session, a practitioner first needs to quiet their mind and ask the animal for permission to communicate (Wijngaarden 2024). Erickson (2014, 1) rephrases this stage as “utilizing simple meditative/contemplative techniques.” Communication must be permitted by the animalsʼ “human guardian” (ibid., 11). TAC catalyses many varying options of information exchange like “clairvoyance (visual), clairaudience (audial), clairsentience (emotional), clairgustance (taste), clairalience (olfactory), and claircognizance (extrasensory)” (Trachsel 2022, 92). Sessions are often conducted remotely, with visual material such as photographs used as a supporting reference point. Communication is also frequently extended to deceased beings (Monvoisin et al. 2024).

The workflows and principles of animal communicators raise questions of falsifiability. Monvoisin et al. (2024) offer bias assessments of TAC studies, showing that all of them include bias levels while assuming only case study positions. Monvoisin and colleagues compared animal communicators’ and regular people's skills of identifying animals as alive or deceased in photographs. They were not able to confirm animal communicators’ expertise since “neither practitioners nor non‐practitioners achieve scores above chance” (ibid., 8). Other critics have pointed to potential researcher bias, particularly in studies involving researchers’ own animals (Sheldrake and Morgana 2003), as well as to difficulties in demonstrating the authenticity and replicability of reported psychic experiences (Erickson 2014).

Although TAC does not constitute a conventional profession, it can be approached as a service occupation in which practitioners must establish credibility towards potential clients. Research on professions, occupations, and organizations has shown that credibility and legitimacy are not necessarily derived from demonstrable technical effectiveness but can be maintained through conformity with institutionalized expectations and practices (Meyer and Rowan 1977). Occupational fields may adopt recognizable structures, routines, and forms of professional presentation that signal legitimacy to relevant audiences (Apkarian 2024), even where the effectiveness of their activities cannot be directly assessed. This perspective is relevant to TAC, where practitioners operate in a market‐based service context while claiming expertise in a domain in which outcomes are unverifiable.

The absence of empirical verifiability in TAC makes the study of broader importance for understanding processes of knowledge acceptance. By investigating this field, the study aims to contribute to the sociology of knowledge by offering an additional perspective on credibility construction under conditions of epistemic uncertainty.

## 2 Methods

### 2.1 Sampling Strategy and Data Corpus

This study is based on a qualitative online document analysis of websites offering TAC services. The aim is to explore how credibility and truth are discursively constructed in the context of spiritual or intuitive service provision. The sample is grounded in a nationwide overview of Germany, with one theoretically relevant case selected for each of the sixteen federal states. Selecting one case per federal state served to introduce structural variation while avoiding an overrepresentation of specific regions or providers with particularly strong online visibility. These cases were identified via a Google search using the query “Animal Communication [Name of federal state]”, and we selected one provider from the first 10 search results. This approach is designed to simulate a typical user search path, approximating how potential clients might seek out such services online.

This territorial sampling was complemented by a theoretical sampling strategy (Glaser and Strauss 1967, 30) to capture contrasts and variations across the field. Cases were purposefully selected to include different thematic foci (e.g., missing animals, end‐of‐life communication, behavioural advice) as well as modes of communication (e.g., in‐person vs. remote sessions). To ensure a diverse and theoretically rich dataset, we defined specific inclusion and exclusion criteria in Table 1:

**TABLE 1:** Inclusion and exclusion criteria.

Inclusion Criteria	Exclusion Criteria
A concrete, bookable service offering in TAC. A dedicated service website. Clear connection to the German‐speaking context (language and/or provider location). Substantive content (background, methods, goals, procedure).	Informational or blog‐only pages. Training or seminar websites without client offerings. Directory listings, forums, or platforms lacking a distinct provider identity. Duplicate entries (e.g., franchise pages, only one version per provider included) Social media profiles without descriptive service information.

The use of theoretical sampling prevents the identification of specific providers. Only publicly accessible content from websites was analyzed, and no interaction with human subjects took place. All providers are pseudonymized, and only indirect quotations are used in the results section, preventing traceability to the original sources. In accordance with copyright regulations and ethical standards, the study adheres to the principles of ‘Fair Use’ (17 U.S. Code § 107), and the materials were used solely for academic purposes. The compilation of the dataset was conducted between November 20 and 25, 2025.

### 2.2 Data Analysis

We follow the principles of Grounded Theory as developed by Glaser and Strauss (1967). The analytical procedure is informed by their logic of theoretical sampling, constant comparison, and theoretical sensitivity. For the practical implementation of the coding process, the structured coding procedures proposed by Strauss and Corbin (1996) were used. We chose this method due to the exploratory nature of the research topic. While the study draws on Strauss and Corbin's (1996) practical coding paradigm, Charmaz’ (2006) constructivist perspective informed the reflexive stance of the analysis.

The analysis follows an inductive coding process, inspired by Strauss & Corbin's (1996, 43–117) three‐step model. The process begins with open coding, where textual material is broken down into discrete units of meaning and assigned abstract labels. During the open coding process, coding units were defined as meaningful segments, which could range from a single word to a full paragraph, depending on how a particular theme was expressed. In the axial coding, these codes are grouped into higher‐level categories, with a focus on their internal relationships. Selective coding then identifies a core category that integrates the various themes and organizes the developing theory. The development from open codes to axial categories and selective coding is summarized in Table 2.

**TABLE 2:** Core categories with axial codes.

Credibility of Telepathic Animal Communication			
Core Categories	Professionalized Presentation	Emotional and Relational Appeal	Strategic Non‐verifiability
**Axial Codes**	Professional Website Features Economic Framing Structured Session Format Therapeutic Terminology Normalization of TAC Referencing Experts References to own TAC Training Additional professional Competencies Biographical Self‐egitimation Legitimation through Experience Advisory Self‐Positioning Explicit Boundaries to Veterinary Care	Familiarization through multimodal presence Emotional Language Anticipated Trust and Relationship Individualized Service Direct and simple Contact Providers Closeness to Animals Positive and emotional Client Reviews Statements from Animals Positive Values and Attributes of the Provider Moral Integrity of the Service Ethical Boundaries	TAC as a magical‐paranormal Realm TAC through Images, Feelings, Thoughts Animal Logic differs: Responses require Interpretation Each Animal has their own Language Animals’ own Will might not be influenced Responses might make Sense later Animals experience Pain differently Deceased Animals provide limited Insight TAC fails if Animal is distracted or refuses TAC fails if Owner is blocked or not ready

Throughout the analysis, theoretical sensitivity (Glaser and Strauss 1967, 47) is maintained by employing generative questions (e.g., What kinds of experiential knowledge are being described?) and systematic comparisons (e.g., How is personal insight framed as evidence?). These techniques enhance reflexivity and support a nuanced interpretation of the data (Strauss and Corbin 1996, 25). The analytical process was facilitated using MAXQDA software.

During the axial coding process, a notable homogeneity across the websites was observed. While new codes continued to emerge at the level of open coding with each additional website, the development of axial categories began to stabilize after approximately ten websites. This indicates that although theoretical saturation (Glaser and Strauss 1967, 61) was not fully achieved at the level of open codes, the emergence of new axial categories was minimal. Thus, the core findings of the study are unlikely to be substantively affected by the inclusion of additional cases.

In line with the principles of Grounded Theory (Glaser and Strauss 1967), we understand comparison as an inherent and continuous aspect of qualitative analysis: data segments are constantly compared with other data, emerging codes with previous codes, and developing categories with one another. This form of comparison is unavoidable, as researchers inevitably draw on prior analytical experiences and existing sensitizing concepts while engaging with empirical material (ibid., 22–24).

## 3 Results

The original data were collected in German. All quotations presented here are English translations by the first author. The original wording was preserved as closely as possible.

The analysis shows that the websites devote a substantial amount of space to outlining a wide range of potential problems that TAC could resolve. On a practical level, TAC is described as helpful in situations of transition and disruption, such as preparing animals for relocation, vacations or surgical procedures. Behavioural issues and physical complaints are also frequently mentioned. Another prominent theme concerns getting in contact with missing animals. On a health‐related level, TAC is framed as enabling problem analyses that go beyond observable symptoms. Particularly striking is the recurring narrative that animals are portrayed as reflecting or absorbing human emotional states. On an emotional and relational level, the websites emphasize themes of grief, guilt and fear, as well as the emotional processing of decisions made on behalf of the animal. TAC is portrayed as a means to cope with the death of an animal, to accompany animals and owners during end‐of‐life care, and to address feelings of guilt related to euthanasia.

TAC is consistently positioned as a tool for coping with problems and restoring balance, while implicitly constructing a problem definition in which animal owners are depicted as not yet fully understanding their animals’ needs. The practice is also framed as enabling a deeper connection between humans and animals.

Providers consistently position themselves as neutral translators, solving problems by mediating between humans and animals, in order to resolve a communicative deficit. This positioning reveals a fundamental tension: while the practice is presented as effective, the mechanisms through which information is received remain empirically inaccessible. Even when outcomes are described as successful, alternative explanations, such as coincidence or sensitivity to contextual cues, cannot be excluded. This epistemic ambiguity is particularly evident in accounts of missing animals, where it remains unclear whether an animal's return can be causally attributed to TAC or would have occurred independently. This underscores that TAC is a spiritual practice whose efficacy cannot be conclusively verified, thereby preparing the ground for a closer analysis of how the credibility of telepathic TAC is produced on provider websites.

**FIGURE 1:**
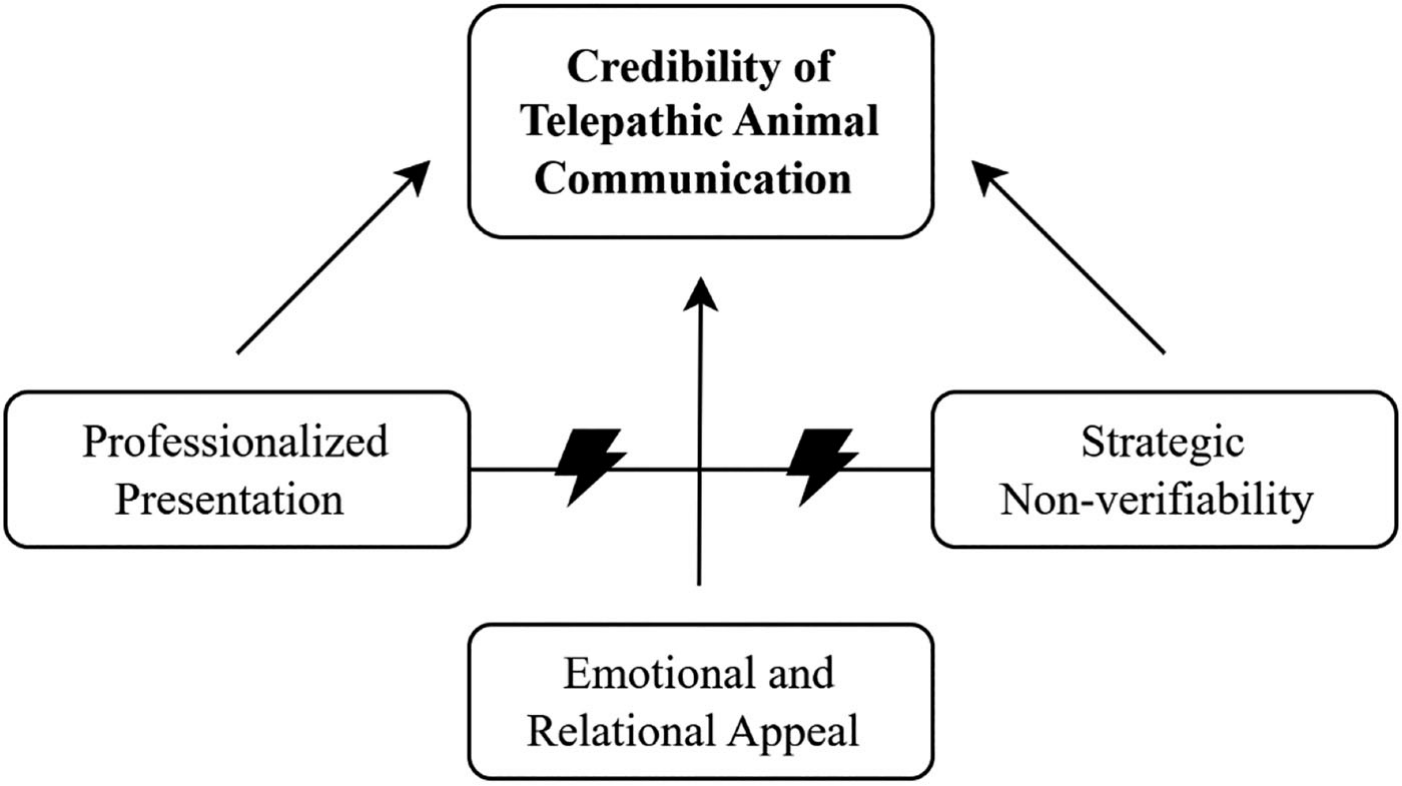
Strategic production and protection of credibility under conditions of non‐verifiability.

The analysis resulted in three interrelated core categories that jointly structure how the credibility of telepathic TAC is constructed on provider websites (Figure 1).

The professionalized presentation of the service refers to the consistent effort to create a structured and serious appearance. This professional framing stands in tension with the non‐verifiable nature of TAC, a tension visually represented in the diagram by lightning symbols. This tension is mediated through the emotional and relational appeal of the service, which should enable clients to accept epistemic uncertainty by foregrounding emotional resonance, relational depth, and subjective meaningfulness.

Non‐verifiability can also serve as a strategy on its own to stabilize credibility. Because communicative processes in TAC cannot be empirically verified and remain open to interpretation, the results are largely insulated from critique or falsification. Together, these dynamics contribute to the stabilization of credibility despite the absence of empirical verification. In the following sections, each of the three categories will be examined in detail.

In the following results section, axial categories are highlighted in italics.

### 3.1 Professionalized Presentation

Professionalized presentation is enacted through the design and structuring of the websites and the way the service is framed. Providers rely on a set of *professional website features* that signal reliability and competence, including contact forms, automated appointment booking systems, FAQ sections, legal information such as imprint and data protection statements, and clearly structured navigation.

Beyond these formal elements, professionalization is further reinforced through *economic framing*. TAC sessions are consistently presented with transparent pricing, typically ranging between approximately 45 and 115 euros per session. Prices are clearly stated and often organized into predefined service packages that specify the offering. For example, packages may include a preliminary consultation, a communication session with a fixed number of questions, a *“bodyscan to identify possible sources of pain,”* a written transcript of the conversation, and a follow‐up discussion. Such standardized pricing models frame TAC as a clearly delimited service and may function as a marker of value and professionalism. This economic framing is complemented by typical market‐oriented features such as discounts for multiple animals or follow‐up sessions, vouchers, and the option to book short‐notice emergency appointments for an additional fee. One provider explicitly links reliability to payment conditions, stating: *“I am available for emergencies at an overtime rate within the next day (after receipt of payment and subject to availability) for you and your animal, because reliability is my top priority.”* This example illustrates how dependability and commitment are directly tied to monetary exchange.

Professionalization is further supported by a *structured session format*. Sessions are generally described as lasting between 60 and 90 minutes and are conducted remotely, most commonly by telephone. Clients are instructed to prepare questions in advance. A recurring requirement is the submission of a photograph of the animal in which the eyes are visible. As one provider explains*, “This image serves as my energetic access, comparable to a telephone number through which I can establish a connection.”*

Professionalization is further accomplished by explicitly situating TAC within established knowledge frameworks and by constructing a coherent professional discourse around the practice. One central strategy is the use of *therapeutic terminology* associated with psychotherapeutic or counselling contexts, such as *“family dynamics,”* *“emotional burden,”* or *“unresolved conflicts.”* By adopting this vocabulary, TAC is discursively aligned with epistemically established helping professions, thereby borrowing legitimacy from adjacent fields. This professional framing is reinforced by the fact that many providers offer seminars that culminate in certificates, positioning TAC as a teachable, formalized skill.

A related strategy concerns the *normalization of TAC: “This is not abnormal.”* Normalization is achieved through claims that telepathy is an innate human ability that most people possessed as children but gradually lost in the context of modern life. It is argued that *“all living beings communicate telepathically.”* Everyday examples of telepathy are invoked, such as thinking of a person shortly before they call. One provider explains TAC in quasi‐scientific terms: *“Every living being (…) possesses an electromagnetic field. (…) This field is connected to the Headsche zones in the skin, which transmit energies from the environment via the spinal nerve roots to the spinal cord and the autonomic nervous system. If you are very sensitive, you can receive information through this field.”* References to professional associations, networks, and international recognition further contribute to normalization, for example through statements such as: *“In the United States, TAC has been recognized for years.”*

Professional credibility is strengthened through *referencing experts* from the field. Most prominently, Penelope Smith is repeatedly cited as a *“pioneer”* of TAC. Other references include Rupert Sheldrake, whose concept of morphic fields is used to explain TAC, as well as various German trainers from whom providers received their education. Literature recommendations further support the impression that TAC is embedded in an existing body of knowledge.

Another recurring strategy concerns the presentation of *additional professional competencies*. Providers frequently list a wide range of further training and skills, such as channeling, energetic healing, Shiatsu, Reiki, homeopathy, nutritional counselling, etc. This multiplicity of qualifications constructs an image of broad expertise, which contributes to professional credibility by suggesting continuous self‐development and interdisciplinary competence.

Biographical continuity, emotional closeness to animals, and morally positive life experiences also enhance credibility. Animal communicative competence is framed as the natural outcome of an empathic life trajectory. This form of *biographical self‐legitimation* is evident in repeated references to early and long‐standing bonds with animals, often described as beginning in childhood. One provider, for instance, reflects on her early experiences by stating: *“Even back then, I felt directly connected to my animal companions and was able to understand them.”*

Closely related is a strategy of *legitimation through experience*. Providers emphasize the number of conducted sessions, long‐standing practice spanning up to several decades, and international activity. Claims such as having conducted *“hundreds of conversations”* position experience itself as a central source of authority. In some cases, this experiential legitimacy is extended into *advisory self‐positioning*, particularly in the context of behavioural issues, where providers describe themselves as offering solution‐oriented guidance based on experience.

Finally, professional credibility is reinforced through explicit boundaries to veterinary care. Statements such as “*Animal communication does not under any circumstances replace veterinary diagnostics and treatment*” clearly delineate the limits of the practice. By referring clients to veterinary or other professional experts, when necessary, providers signal reflexivity and responsibility, thereby further stabilizing their professional self‐presentation.

### 3.2 Emotional and Relational Appeal

*Familiarization through multimodal public presence* enables potential clients to develop a sense of personal connection and trust toward the provider. Through a wide range of publicly accessible materials, such as introductory videos, YouTube channels, social media profiles, interviews, television appearances, books, blog posts, or free introductory workshops, providers make themselves visible across multiple formats. This multimodal presence allows observers to see how practitioners present their work, thereby fostering familiarity and trust.

*Emotional language* is a key strategy through which TAC providers cultivate trust and relational closeness on their websites. Themes are frequently presented in a vivid and affective manner, emphasizing the emotional depth of TAC. One provider describes: *“Animal communication is the bridge between human and animal, a quiet yet powerful exchange full of feelings.”* The animals themselves are depicted as experiencing deep emotional responses, for example, *“Joy at being perceived and the ability to express long‐held gratitude.”* Emotional language, such as *“I look forward to meeting you and your four‐legged friend,”* creates a heartfelt tone that frames the service as intimate and trustworthy.

Closely related is the deliberate cultivation of *anticipated trust and relationship*. Providers frequently address clients directly, employing a familiar tone and describing interactions as collaborative: *“Let's build a deeper connection with your animal together.”* Websites also often include personal narratives, sharing the practitioner's journey into TAC, experiences with their own pets, or their broader life context, including family, hobbies, and values. One illustrative example reads: I like *“Animals, nature, long walks, the sea, summer, reading, writing, painting, meditating (…).”* These disclosures function like a personal profile, allowing potential clients to anticipate a trusting relationship with the provider.

Websites frequently emphasize that TAC is an *individualized service*. For example, one provider states: *“Every being is unique, and so is every conversation with an animal.”* This framing communicates that the practitioner takes the time to respond to the specific needs and circumstances of both the animal and its owner. By highlighting this individualized approach, providers signal that the service is flexible and client‐centred, reinforcing emotional appeal.

*Direct and simple contact* is another mechanism through which providers build trust and closeness. Clients are encouraged to contact the practitioner directly, without intermediaries such as a receptionist: *“You can also easily check and book appointments for animal communication sessions or courses right here via the blue button”*. Communication channels include phone, WhatsApp, email, and direct contact forms, often complemented by options to book sessions or subscribe to newsletters. Publicly shared contact data convey transparency and availability. This direct access reinforces the impression of an approachable and personally invested practitioner.

The *provider's closeness to animals* is frequently emphasized. One provider describes herself as a *“dog mom,”* while others note lifelong experience with pets or extended periods living closely with animals. This enduring personal connection could foster client confidence in their competence to offer TAC services.

*Positive and emotional client reviews* emerge as a central strategy of experiential legitimation. Across the material, testimonials consistently narrate tangible outcomes and emotionally meaningful experiences. Reviews frequently describe behavioural or well‐being changes in animals, such as pets coping better with stressful situations like New Year's Eve: *“My two darlings had a relaxed New Year's Eve like they haven't had in years.”* Clients emphasize feeling emotionally closer to their animals after the session: *“Through your communication, I have grown even closer to my dogs* *.”* Others highlight reassurance in difficult decision‐making processes, including health‐related choices or determining the appropriate moment for euthanasia, as well as support in grief work following illness or loss: *“The comfort is so great and wonderful that it truly makes the grief more bearable!”* Particularly striking are outcomes, such as missing animals being found or returning home: *“Through the communication, our dog fought and safely returned home after eight days.”* Credibility is further intensified through accounts that merge transformed skepticism: clients stress that communicators addressed information they could not have known in advance and that they recognized their animal's personality as accurately reflected in the conversation. Many reviews foreground the empathic and relational qualities of the providers.

A related phenomenon concerns the inclusion of *statements from animals*. These also function as a legitimating strategy by offering compact success stories from a non‐human perspective. For example, a sheep announces the imminent birth of her lamb, which is confirmed the following day; a dog affected by construction noise asks for its bed to be moved to reduce stress; or a deceased cat describes: *“Without a body, you have completely different possibilities again.”* Through such animal statements, the functioning of the service is implicitly confirmed by the animals themselves.

Another feature is the emphasis on *positive values and attributes of the provider*. Providers highlight a strong sense of responsibility and awareness of the emotional weight of their work. TAC is presented as a *“heartfelt practice,”* motivated by joy and the desire to *“help both humans and animals.”* Empathy is repeatedly highlighted and seems to function as a core professional competence, expressed in statements such as translating animals’ messages *“with great empathy”* or helping *“with passion and heart”* to foster mutual understanding and trust. Providers also highlight personal qualities like *“groundedness, idealism, client closeness, responsiveness, and humour.”* This value‐driven self‐presentation reinforces credibility by combining moral integrity with relational competence.

A key aspect of the *moral integrity of the service* is established through reference to ethical guidelines, notably the Penelope Smith Code of Ethics, which frames TAC as a respectful and mindful practice. Providers emphasize respect for animals, clarify that they distinguish between the animal's messages and their own interpretations, and make clear that decisions remain with the client. Engagement in animal welfare further reinforces the moral grounding of the service.

This moral foundation is further reinforced through *ethical boundaries*, emphasizing clear limits to what TAC can and cannot achieve. Providers explicitly state that services are not a form of healing, psychological counselling, or a substitute for training or veterinary care. Critical decisions, such as those related to surgery or euthanasia, remain with the client, and animals are never coerced or manipulated. Sessions are treated as confidential, and no information about third parties is shared.

### 3.3 Strategic Non‐Verifiability

The analysis suggests that non‐verifiability does not operate as a barrier to the legitimation of TAC. One might assume that the inability to empirically verify communicative outcomes would undermine credibility. However, the analysis revealed a counterintuitive pattern that emerged inductively from the data: non‐verifiability is a resource for credibility. Through the articulation of multiple, mutually accommodating interpretive possibilities, communicative outcomes are rendered resistant to falsification, thereby stabilizing the credibility of the service.

A central pattern in the data is the recurrent depiction of *TAC as a magical‐paranormal realm*. It is described as an *“own world”* and associated with notions such as *“intuition,”* *“fascination,”* *“adventure,”* and *“paranormal perception.”* Several providers explicitly state that TAC cannot be fully understood through rational explanation but *“must be experienced firsthand.”* Explanatory references, for example, to a morphic field, further contribute to this framing while remaining vague and scientifically unverified. Through this positioning, TAC is discursively separated from everyday forms of knowledge, which limits the relevance of external verification.

Communication is said to occur through inner “*images*, *feelings*, *and thoughts,”* with messages being received on an internal cognitive or intuitive level. Such descriptions locate the communicative process in an invisible and subjective realm that is inaccessible to external observation. As a result, the plausibility of communicative experiences cannot be contested.

A particularly salient aspect of this pattern is the repeated emphasis that *animal logic differs* and that *responses require interpretation*. Messages from animals are not presented as direct or unambiguous statements but as impressions that must be contextualized and interpreted by the communicator and the owner. For example, when animals are described as expressing fear, these emotions may be interpreted as the animal's own or as reflections of the owner's emotions. Similarly, animals can perceive and evaluate situations in fundamentally different ways. This interpretive openness becomes especially visible in accounts of missing animals. Locations communicated by the animal are often described in abstract terms, such as colors, images, or vague spatial impressions, which then require further interpretation to be translated into concrete places: *“Animal logic is different from human logic. (…) They can often only reproduce fragments, have no chronological order, leave out processes that are important to us, are in shock, or, out of fear of enemies, simply shut down their energy. Sometimes there are meaningful things among them that support us; sometimes a great deal of imagination is needed to relate them to real events (…). Animals in fear can also fantasize (…). This is why it may also be the case that I tell you what feels like very little or nonsense, even though it is the animal's truth at that moment.”*

In addition, providers frequently emphasize that “*each animal has their own language.”* This individuality further expands the interpretive space, as communicative outcomes cannot be assessed against standardized expectations.

References to the *“animal's free will”* introduce an additional layer of uncertainty. Even if TAC is described as successful, the *animal's own will might not be influenced*. Therefore, subsequent changes in behavior are said to depend on the animal's own decisions and may or may not occur: *“You can, of course, explain a wish to your animal, but whether it wants this is ultimately its own decision.”* Consequently, the success or failure of TAC remains difficult to determine.

The claim that *responses might make sense later* further reinforces interpretive openness: *“Some statements may not be clear or understandable at first, and their meaning may only become apparent over time.”* If a communicative outcome does not appear meaningful to the animal's owner, its relevance can be deferred to an unspecified future moment. Through this potential temporal postponement, the plausibility of the response is preserved.

Interpretive openness also extends to the attribution of pain and physical states, as *animals experience pain differently*. In the context of missing animals, one provider emphasizes that an animal's perceived condition does not necessarily correspond to its physical state. A missing animal may be described as feeling well even if it is already deceased, as it may not be aware of its separation from the body: *“Some animals show how comfortable they feel and, because of the shock, have not yet realized that they have left their body and that it is lying there lifeless.”* Such accounts allow communicative statements to remain coherent regardless of outcomes. Whether the animal is later found alive or not, previously articulated descriptions can be retrospectively aligned with the situation.

In communication with deceased animals, interpretive limits are further reinforced by the claim that *deceased animals provide limited insight*: “*The deceased give us a small glimpse through the keyhole into a world hidden from us*.” Providers emphasize that deceased animals are not allowed to share certain forms of knowledge, for example regarding the nature of the afterlife or insights gained after death.

Potential failures of TAC are explained through factors that remain inaccessible to verification. Communication is said to *fail if the animal is distracted or refuses* to engage: *“If the animal is too excited, the communication may become somewhat less clear,”* or if the *owner is blocked or not yet ready* to receive certain information: *“It is us humans who resist.”* As neither the animal's internal state nor the owner's readiness can be independently assessed, unsuccessful outcomes cannot be attributed to the provider.

A focused comparison of TAC websites with selected websites offering dentistry, psychotherapy, car repair, and electrical services further indicated that strategic non‐verifiability is distinctive to TAC. While professionalized presentation and relational appeal were also present across these service domains, credibility was more strongly anchored in externally observable and verifiable outcomes. In TAC, the absence of external verification creates a specific reliance on interpretive flexibility as a means of stabilizing credibility. This interpretive openness enables outcomes to remain adaptable and retrospectively coherent, thereby supporting the credibility of the practice.

## 4 Discussion

The findings suggest that credibility in TAC is constructed through a combination of professional self‐presentation, emotional trust‐building, and the strategic management of non‐verifiability. While the first two mechanisms resonate with established research on credibility and trust, the third extends existing discussions by highlighting how the absence of verification may itself become a resource for maintaining credibility. The following discussion examines each category in relation to the broader literature and considers its implications for the sociology of knowledge.

### 4.1 Credibility and Professionalized Presentation

One of the central challenges of non‐verifiable knowledge claims is that their credibility cannot be established through direct empirical confirmation. To generate trust in such claims, professionalized presentation constitutes an alternative mechanism that may nevertheless lead audiences to perceive otherwise unverifiable statements as plausible and legitimate.

Across the analyzed websites, practitioners frequently highlight qualifications, certifications, years of experience, and professional biographies. From a sociological perspective, these practices can be understood as forms of impression management through which actors seek to convey competence and legitimacy (Goffman 1959; Pillemer 2024). In the context of online websites, the concept of virtual impression management can be applied, which also includes, for example, the aesthetic dimension of a website (Blunden and Brodsky 2024). Through the adoption of visual and textual conventions associated with established professional services (Flanigan and Metzger 2007), practitioners present themselves as knowledgeable experts despite the absence of institutional regulation or scientific validation of their claims. More broadly, the findings indicate that professionalization itself functions as a symbolic resource for credibility construction.

This observation is consistent with research on credibility assessment and trust formation. Studies suggest that individuals frequently rely on heuristic cues when evaluating information that is difficult to verify directly. Rather than assessing the validity of claims themselves, audiences often infer credibility from indicators such as professionalism, expertise, consistency, and presentation quality (Metzger et al. 2010; Sundar 2008). In contexts characterized by epistemic uncertainty, such cues may become particularly influential because they provide recognizable signals of competence and reliability.

A similar argument has been made in marketing and consumer research, where trust is regarded as a prerequisite for the acceptance of intangible services (Morgan and Hunt 1994). Because the quality of such services often cannot be evaluated prior to consumption, potential clients rely on signals that communicate expertise and professionalism (Flanigan and Metzger 2007). TAC represents a particularly pronounced example of this challenge, as clients are not only unable to assess service quality beforehand but are also largely unable to independently verify the underlying knowledge claims. Professionalized self‐presentation may therefore serve as an important mechanism for reducing uncertainty and establishing trust.

Similar patterns have also been observed in other contexts involving non‐verifiable knowledge claims, such as spiritual conspiracy theories. In a qualitative study of YouTube content related to the Prison Planet conspiracy theory, Barzen (2025) found that credibility was constructed through the perceived authority and trustworthiness of the content creator. In the presentation of his content, the creator employed structured argumentation, references to expertise, and a high degree of transparency regarding his reasoning processes, while simultaneously framing his interpretations as self‐evidently logical, despite the absence of empirical evidence. The present findings suggest that comparable mechanisms may operate within TAC, indicating that professionalized credibility construction is not limited to market‐based services but may represent a broader strategy through which actors establish legitimacy in contexts where empirical verification is unavailable.

### 4.2 Credibility and Emotional/Relational Appeal

Under conditions of non‐verifiability, trust in the communicator becomes particularly important. Rather than evaluating the validity of the knowledge claim itself, audiences may rely on the emotional and relational proximity established by the person presenting it. The findings suggest that TAC practitioners support the credibility of their knowledge and abilities by fostering emotional connections with potential clients.

This observation can be connected to research on virtual impression management. Blunden and Brodsky (2024) argue that actors in digital environments strategically manage impressions through a variety of online self‐presentation practices. Beyond a professional design, this also includes self‐disclosure, namely the communication of personal experiences, emotions, values, and biographical information. Self‐disclosure can increase perceptions of authenticity and interpersonal closeness, thereby strengthening trust in the communicator. This pattern was clearly visible in the analyzed websites, where practitioners frequently shared personal stories about their relationships with animals, their childhood, moments of personal transformation, or experiences that allegedly led them to discover their communicative abilities. Such personal disclosures may reduce social distance and encourage potential clients to view practitioners as sincere and trustworthy individuals rather than merely service providers.

The importance of relational trust has also been emphasized in marketing and service research. Relationship marketing theory proposes that successful service relationships depend on the development of trust between provider and client (Morgan and Hunt 1994). Similarly, Berry (1995) argues that relationship building represents a key strategy in service contexts, where trust and emotional connection can be as important as technical competence. These observations can further be interpreted through Hochschild's (2007) concept of emotion management. Hochschild argues that emotional expressions within service interactions are often shaped by socially expected feeling rules and can become part of professional practice. Displays of empathy, compassion, and emotional understanding are not necessarily spontaneous but may also function as communicative resources. The findings suggest that TAC practitioners actively cultivate such relationships by emphasizing their role as caring, empathetic, and emotionally invested individuals who are deeply concerned with the well‐being of both animals and their owners.

Again, a similar pattern can be observed in the context of spiritual conspiracy theories. Barzen (2025) found that credibility was strengthened through the creation of perceived intimacy between content creators and audiences. Direct forms of address, the sharing of personal experiences, and the presentation of one's own intellectual journey contributed to the construction of trust and epistemic authority. The present findings indicate that comparable mechanisms may operate within TAC, where credibility is established through the cultivation of a personal relationship with potential clients.

An additional dimension of relational credibility construction is provided by customer reviews. In online environments, credibility is often co‐produced through the evaluations of others. Research has shown that user‐generated reviews function as important credibility cues, particularly when they contain detailed and emotionally rich accounts of personal experiences (Wiedmann et al. 2011). In the present material, testimonials frequently emphasized emotional relief, feelings of being understood, or positive transformations in the relationship between humans and animals. Such accounts do not empirically validate TAC but may function as affective evidence that reinforces trust and stabilizes practitioners’ credibility.

### 4.3 Credibility and Strategic Non‐Verifiability

One particularly noteworthy finding of this study is the role of strategic non‐verifiability in the construction of credibility. While professionalized self‐presentation and emotional‐relational appeal have been documented across a wide range of contexts (e.g., Nazzal 2024; Vrtana and Krizanova 2023), the present findings suggest an additional mechanism that has received considerably less attention. TAC practitioners appear to actively mobilize non‐verifiability itself as a credibility‐enhancing resource. By maintaining interpretive flexibility and limiting opportunities for clear falsification, knowledge claims can remain plausible even when outcomes are ambiguous or contradictory.

The findings can also be situated within Meyer and Rowan's (1977) concept of the “rationalized myth,” referring to institutionalized structures, procedures, and forms of expertise that are socially defined as rational and legitimate, independent of their demonstrable technical effectiveness. Schools and hospitals provide illustrative examples: the outcomes of teaching or medical treatment cannot be fully assessed independently of the many factors that shape them. Qualifications, standardized procedures and other institutionalized markers of expertise provide recognizable grounds for confidence in these organizations. Meyer and Rowan describe a “logic of confidence and good faith” (p. 357), explaining how confidence can be maintained despite limited possibilities for directly evaluating organizational performance. They further note that organizations may “minimize inspection and evaluation” (p. 359), thereby reducing the extent to which their technical performance is subjected to direct scrutiny. The present findings point to a related but more specific mechanism in TAC: practitioners maintain interpretive flexibility that limits the possibility of disconfirmation. Strategic non‐verifiability may therefore contribute to the stabilization of credibility precisely because independent verification remains difficult.

The comparison between sociological theories and conspiracy theories suggests that verifiability constitutes a key point of divergence for accepting theories as credible or not (Barzen 2024). While sociological theories are open to empirical scrutiny, conspiracy theories often rely on claims for which crucial evidential components are absent or inaccessible. Although such missing information may later be supplemented by whistleblowers, these claims typically remain unverifiable at the time of their circulation due to their reliance on concealed or inaccessible domains. This example illustrates that verifiability may function as a critical threshold at which knowledge becomes socially accepted and at which social reality itself is constituted.

The findings also point to a potential blind spot in existing theories of trust and knowledge. Classical sociological accounts, ranging from Durkheim (1893) and Simmel (1900) to Luhmann (1968), as well as more recent approaches to epistemic trust and credibility (Eyal et al. 2024; Schilke et al. 2021), have primarily focused on how trust is established under conditions of uncertainty. These perspectives typically assume that the object of trust remains, at least in principle, open to future verification or disconfirmation. The present findings suggest a different configuration: trust is placed in claims that are structurally resistant to verification. In such contexts, trust may not function as a temporary substitute for missing information but as a more enduring mechanism through which unverifiable knowledge is stabilized and maintained.

Beyond epistemic considerations, non‐verifiability may also function as a mechanism through which asymmetries of power are maintained. When access to information is restricted or when claims cannot be independently verified, the authority to define “what is true” shifts toward those who control interpretation. This dynamic has been discussed in sociological accounts of power and knowledge (Foucault 1977), where regimes of visibility and discourse structure what can be known. Similar mechanisms have been identified in contexts of high dependency and controlled information environments, such as studies on coercive group structures (Lalich 2004), where limited access to external verification contributes to the stabilization of authoritative narratives.

From a broader epistemological perspective, the strategic use of unverifiability extends beyond the specific field of TAC. In qualitative research traditions informed by social constructivism (Berger and Luckmann 1966) and post‐positivism (McLennan 2006), knowledge production likewise acknowledges the situated, interpretive, and non‐replicable nature of findings (Kim 2025; Pervin and Mokhtar 2022). Concepts such as the co‐construction of meaning and the influence of researcher subjectivity imply that results are not objective or universally verifiable (Jacoby and Ochs 1995; Kalu 2019). While qualitative research approaches rely on systematic and transparent procedures, they nonetheless operate within an epistemic framework in which multiple interpretations can coexist and “truth” remains, to some extent, perspectival. These considerations invite broader reflection on how credibility and trust are established in contexts where knowledge claims cannot be fully separated from processes of interpretation.

The presented insights can contribute to the sociology of science by questioning the nature of scientific knowledge and the approval of the very same through different social actors. In particular, the finding of strategic non‐verifiability points towards a promising avenue for future research on how scientific credibility is established and maintained when claims cannot be straightforwardly verified or falsified. Strategic non‐verifiability could thereby be further examined in dialogue with Science and Technology Studies perspectives on the social production and stabilization of scientific knowledge (Collins 1985; Latour and Woolgar 1986), as well as with work on epistemic cultures (Knorr Cetina 1999) and the co‐production of science and social order (Jasanoff 2004). As Collins (1985) notes, aspects as small as “differing vocabulary and differing conceptual landscape[s] of scientists” (p. 122) can be highly significant for widespread knowledge acceptance. Thus, strategic non‐verifiability can be viewed as a reminder not to simply accept information at first glance.

Beyond the specific case of TAC, the findings point to the broader analytical value of studying heuristic extreme cases of knowledge production. Because the claims made within TAC are particularly resistant to independent verification, mechanisms of credibility construction become unusually visible. TAC may be understood as an empirical field that allows processes of trust formation, legitimacy construction, and the stabilization of knowledge claims to be examined in a particularly pronounced form. Theoretical contributions developed from such cases may extend beyond the immediate empirical field and contribute to the development of middle‐range theories of credibility construction under conditions of non‐verifiability. Similar dynamics may also be found in other domains characterized by limited possibilities for external verification, such as spiritual and religious knowledge claims. Exploring such cases may therefore represent a particularly fruitful avenue for future research in the sociology of knowledge, as they provide opportunities to further refine theoretical understandings of how credibility is constructed and maintained when empirical verification is unavailable.

Future research could investigate how strategic non‐verifiability operates across different epistemic contexts and how actors use interpretive openness to negotiate the credibility of scientifically framed claims. It may also investigate whether strategic non‐verifiability is also employed in other domains of knowledge production characterized by limited possibilities for empirical verification. Such studies could help assess the transferability of this concept beyond TAC and evaluate its broader theoretical relevance for understanding how credibility is constructed and maintained in contexts of epistemic uncertainty.

## 5 Limitations

The sampling strategy combines territorial and theoretical considerations but does not aim at representativeness. The focus on the first ten Google search results reflects algorithmically shaped visibility. Providers with limited online presence or alternative modes of client acquisition may therefore be underrepresented. The analysis is also based on publicly accessible website material. While this allows for the examination of discursive self‐presentations, it does not capture offline practices or client experiences. Although axial‐level saturation was reached, open coding continued to generate additional refinements. The inclusion of further cases might have expanded or nuanced individual categories. Finally, the findings are shaped by the researcher's subjective perspective. Although reflexive strategies and constant comparison (Glaser and Strauss 1967) were employed throughout the process, alternative analytical perspectives may have led to different emphases.

## 6 Conclusion and Outlook

The study examined how credibility of non‐verifiable knowledge is socially constructed in the empirical context of TAC. We have shown that credibility on TAC websites is constructed through a combination of professionalized presentation and emotional‐relational appeal. Providers frame their services in ways that signal competence, structure, and procedural clarity, while simultaneously establishing proximity and trust through affective language and relational address. A particularly distinctive and novel finding is the strategic use of non‐verifiability. Rather than presenting non‐verifiability as a limitation, providers often foreground the interpretive flexibility of communication results. By framing outcomes as open to different readings, potential contradictions are pre‐emptively absorbed, thereby stabilizing credibility claims in the absence of conventional evidence. These findings are especially relevant for the sociology of knowledge, as they highlight how legitimacy can be constructed beyond classical models of scientific validation. The study contributes to a still underexplored field by demonstrating how alternative forms of knowledge mobilize their own credibility regimes.

## Authors Contribution

Annika Barzen: conceptualization, methodology, data analysis, writing—original draft, writing—review and editing. Dylan Göpfert: data collection, writing—original draft (parts of the introduction and discussion sections).

## Funding

This research received no external funding. Both authors were affiliated with the Institute of Sociology at Chemnitz University of Technology at the time of writing.

## Ethics Statement

This study is based exclusively on publicly accessible websites and did not involve interaction with human participants. Formal ethical approval was therefore not required. All material was anonymized, and no identifiable information is reported.

## Conflicts of Interest

The authors declare no conflicts of interest.

## Generative AI Statement

ChatGPT (OpenAI, GPT‐5.3; https://chatgpt.com) as well as DeepL Translator (DeepL SE, v2, https://www.deepl.com/de/translator) were used for translation and language editing only. The AI tools were last used on May 8, 2026.
